# Supplementary material for: Boosting classical and quantum nonlinear processes in ultrathin van der Waals materials
Source: Nat Commun. 2025 May 29;16:4987. doi: 10.1038/s41467-025-58449-3 (PMC12122887; doi:10.1038/s41467-025-58449-3)
Supplement: Supplementary file 1 — Supplementary Information [file 41467_2025_58449_MOESM1_ESM.pdf]

Supplementary Information for "Boosting classical and quantum nonlinear processes in ultrathin van der Waals materials"

Xiaodan Lyu[1,2,†], Leevi Kallioniemi[1,†], Hongbing Cai[3], Liheng An[1], Ruihuan Duan[4], Shuin Jian Wu[5], Qinghai Tan[6], Chusheng Zhang[1], Ruihua He[7], Yansong Miao[7], Zheng Liu[4], Alexander Ling[5,8], Jesus Zúñiga-Perez[1,2,*], Weibo Gao[1,2,5,9,10,*]

[1]Division of Physics and Applied Physics, School of Physical and Mathematical Sciences, Nanyang Technological University; Singapore, 637371, Singapore

[2]Majulab, International Research Laboratory IRL 3654, CNRS, Université Côte d'Azur, Sorbonne Université, National University of Singapore, Nanyang Technological University, Singapore, Singapore

[3]Department of Physics, University of Science and Technology of China, Hefei, China

[4]School of Materials Science and Engineering, Nanyang Technological University, Singapore, 639798, Singapore

[5]Centre for Quantum Technologies, Singapore, Singapore

[6]School of Microelectronics, University of Science and Technology of China, Hefei, China

[7]School of Biological Sciences, Nanyang Technological University, Singapore, Singapore

[8]Department of Physics, National University of Singapore, Blk S12, 2 Science Drive 3, 117551, Singapore

[9]School of Electrical and Electronic Engineering, Nanyang Technological University, Singapore, Singapore

[10]Quantum Science and Engineering Centre (QSec), Nanyang Technological University, Singapore, Singapore

† These authors contributed equally to this work.

**Corresponding author. Email: jesus.zuniga@ntu.edu.sg; wbgao@ntu.edu.sg*

Supplementary Figures

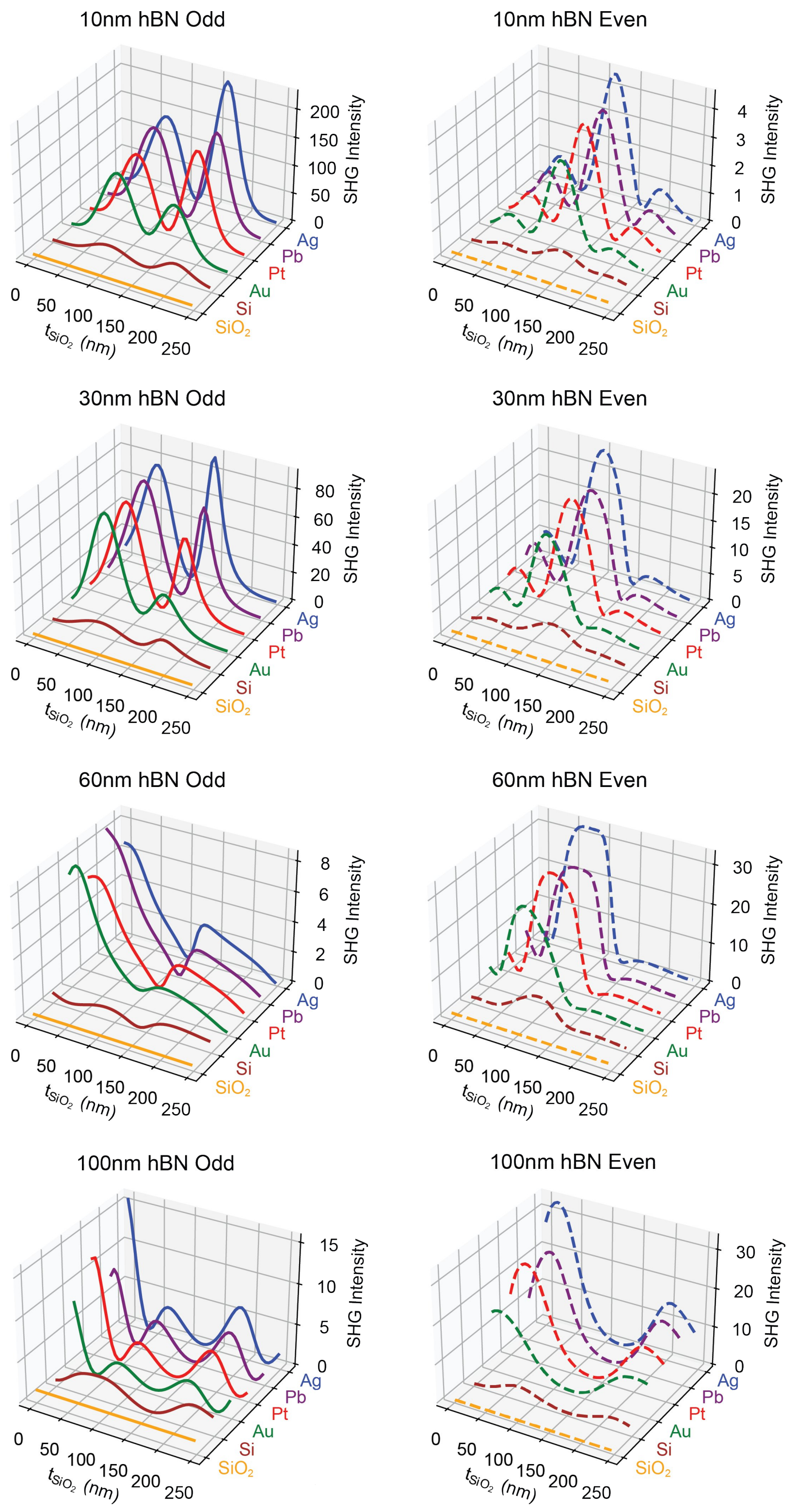

Supplementary Figure 1 | SHG responses from material/SiO_2/X, where X represents Si, various metals and fused silica at various h-BN thicknesses.

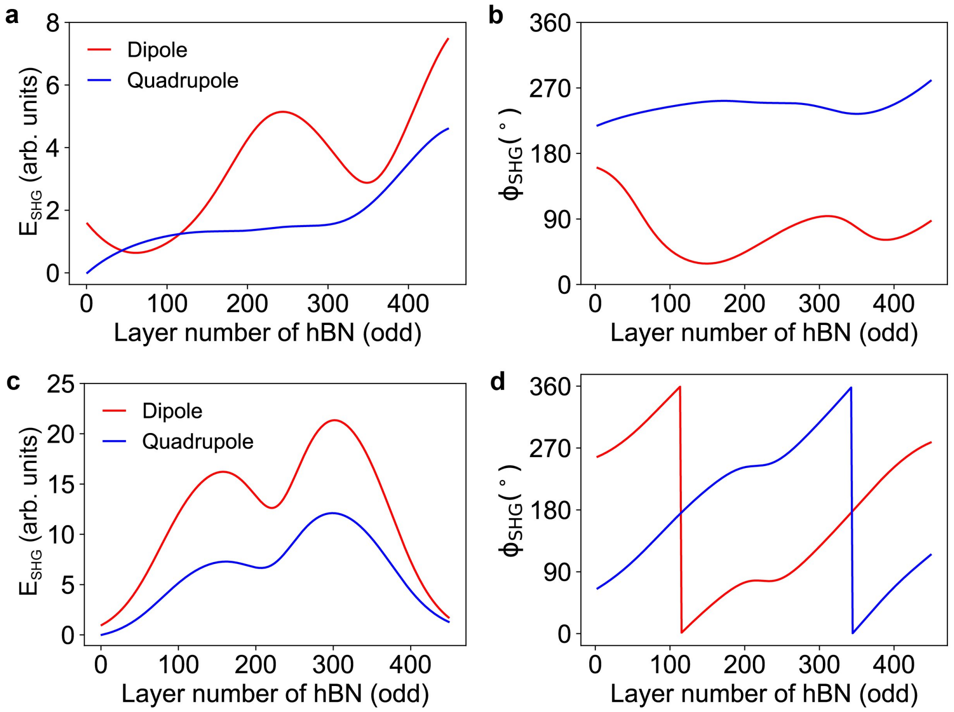

Supplementary Figure 2 | Dipolar and quadrupolar contributions for odd-parity h-BN. **a,c,** The magnitude of SHG electric field from dipole and quadrupole contributions, as a function of the odd total layer number of h-BN on SiO_2/Si (a) and gold (c) substrate. Results for even layers not shown, since the dipole moment vanishes. **b,d,** The phase of SHG electric field from dipole and quadrupole contributions, as a function of the odd total layer number of h-BN on SiO_2/Si (b) and gold (d) substrate.

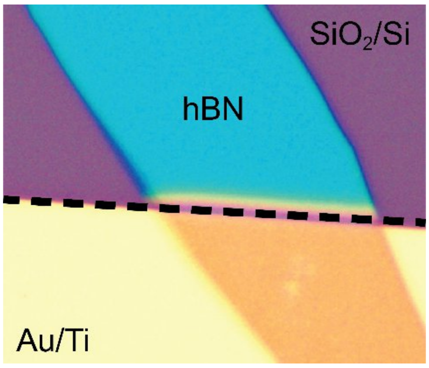

Supplementary Figure 3 | Optical image of h-BN flake deposited on gold and SiO_2/Si.

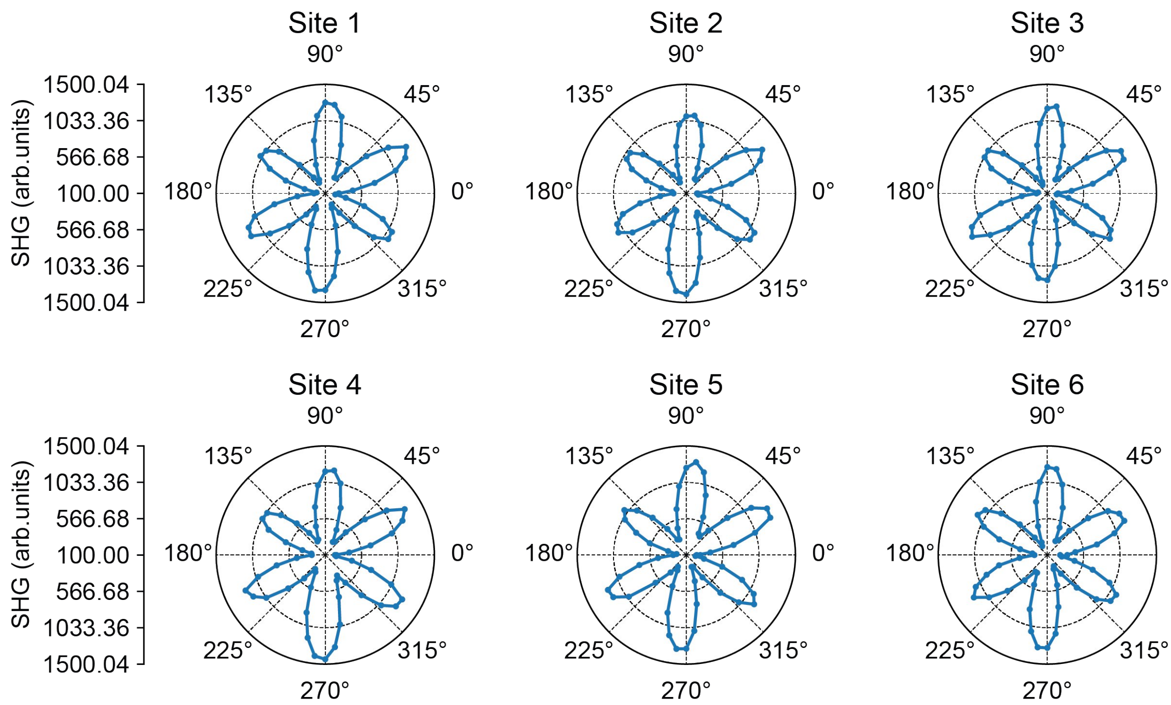

Supplementary Figure 4 | SHG measurement from 36 nm h-BN on Au film.

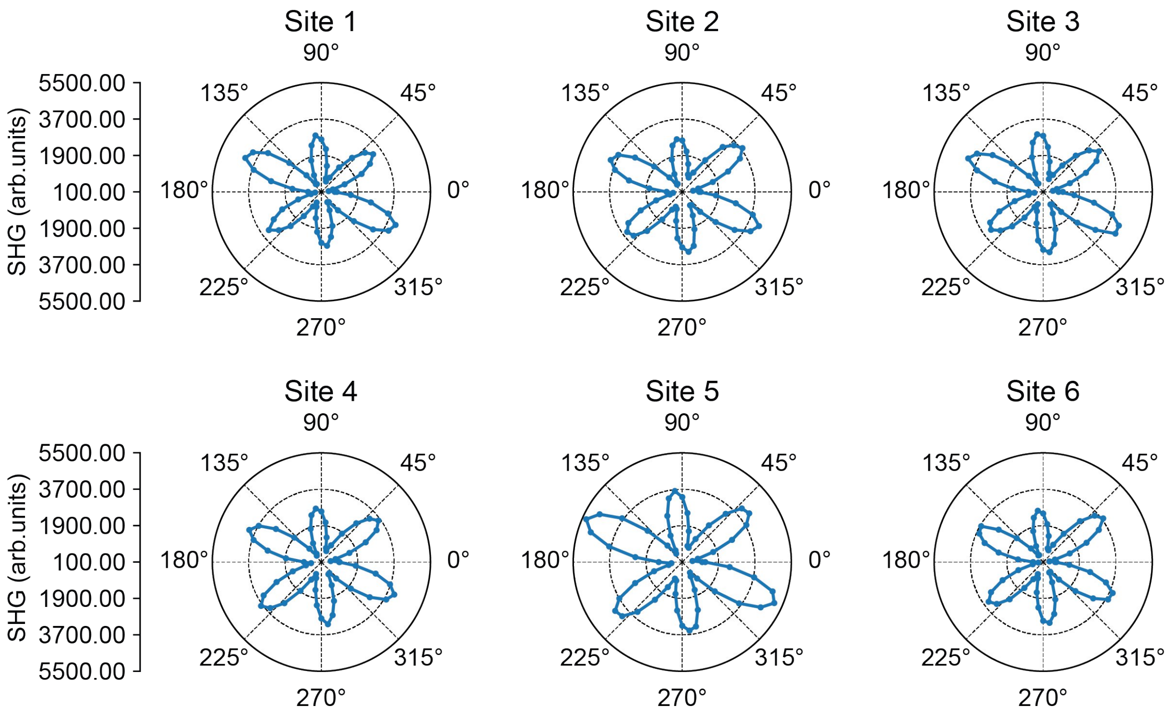

Supplementary Figure 5 | SHG measurement from 65 nm h-BN on Au film.

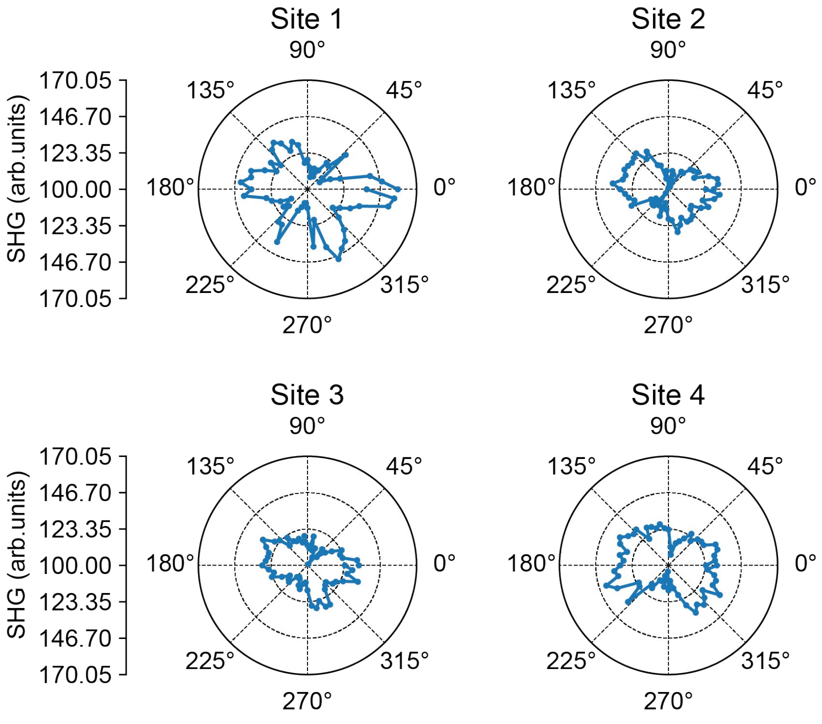

Supplementary Figure 6 | SHG measurement from Au film.

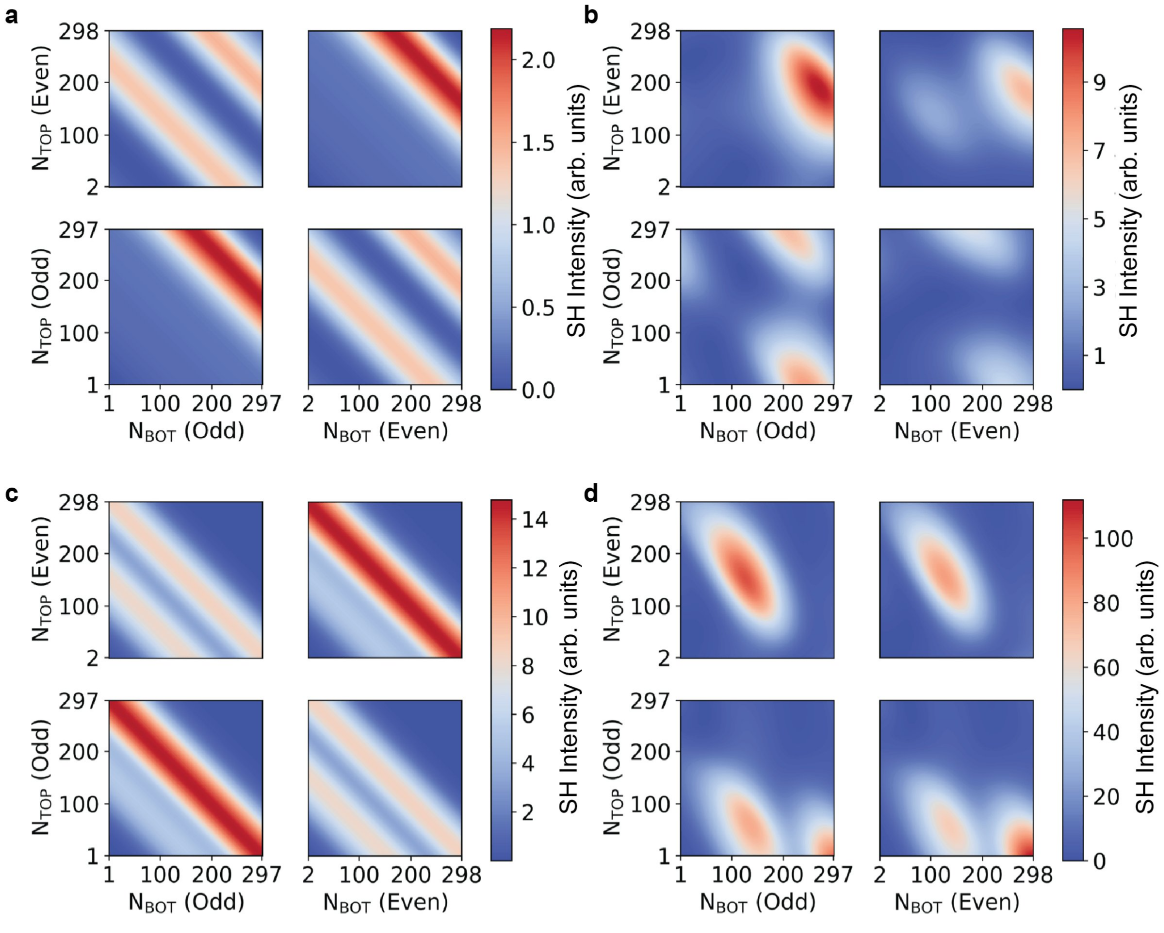

Supplementary Figure 7 | Simulations of SH intensities of twisted h-BN homostructures on different substrates. a, SH intensity for h-BN flakes with AA' stacking order on SiO_2/Si substrates, showing dependence on the parity of the total layer number. **b,** SH intensity for h-BN flakes with AB stacking order on SiO_2/Si substrates, demonstrating the influence of the parity of the top layer. **c,** SH intensity for h-BN flakes with AA' stacking order on gold substrates, exhibiting a shift in maximum points attributed to changes in the incident electric field. **d,** SH intensity for h-BN flakes with AB stacking order on gold substrates, indicating a dependence on flake symmetry, incident electric field distribution, and layer numbers in the assembled structure.

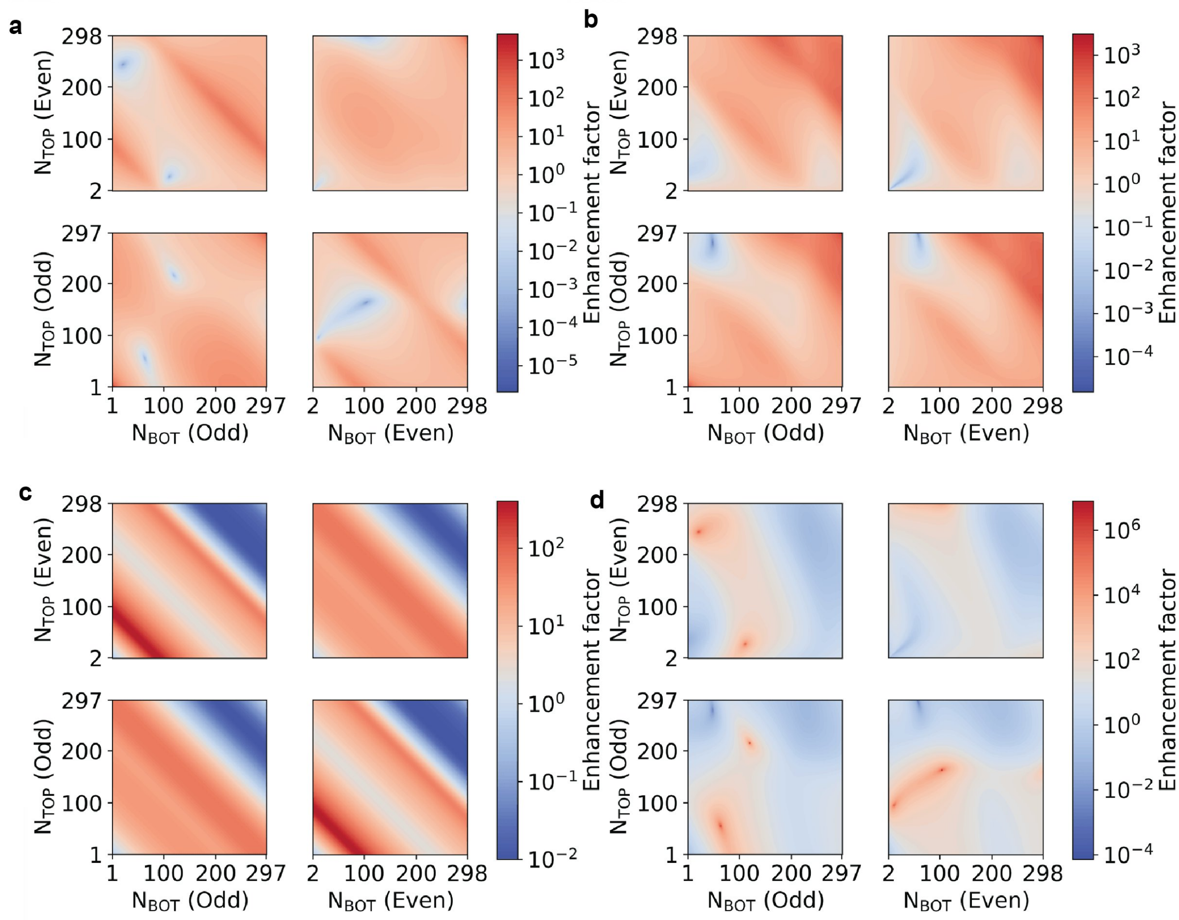

Supplementary Figure 8 | Simulations of the enhancement factor for twisted h-BN homostructures on different substrates. a, Enhancement factor for h-BN flakes with AB stacking order compared to AA' stacking order on SiO_2/Si substrates, revealing a higher

enhancement for odd total layer numbers. The maximum enhancement occurs at a total layer number of 375. **b,** Enhancement factor for h-BN flakes with AB stacking order compared to AA' stacking order on gold substrates. **c,** Enhancement factor for h-BN flakes with AA' stacking order on gold substrates compared to SiO_2/Si substrates, indicating a significant dependence on the parity of the total layer number. **d,** Enhancement factor for h-BN flakes with AB stacking order on gold substrates compared to SiO_2/Si substrates.

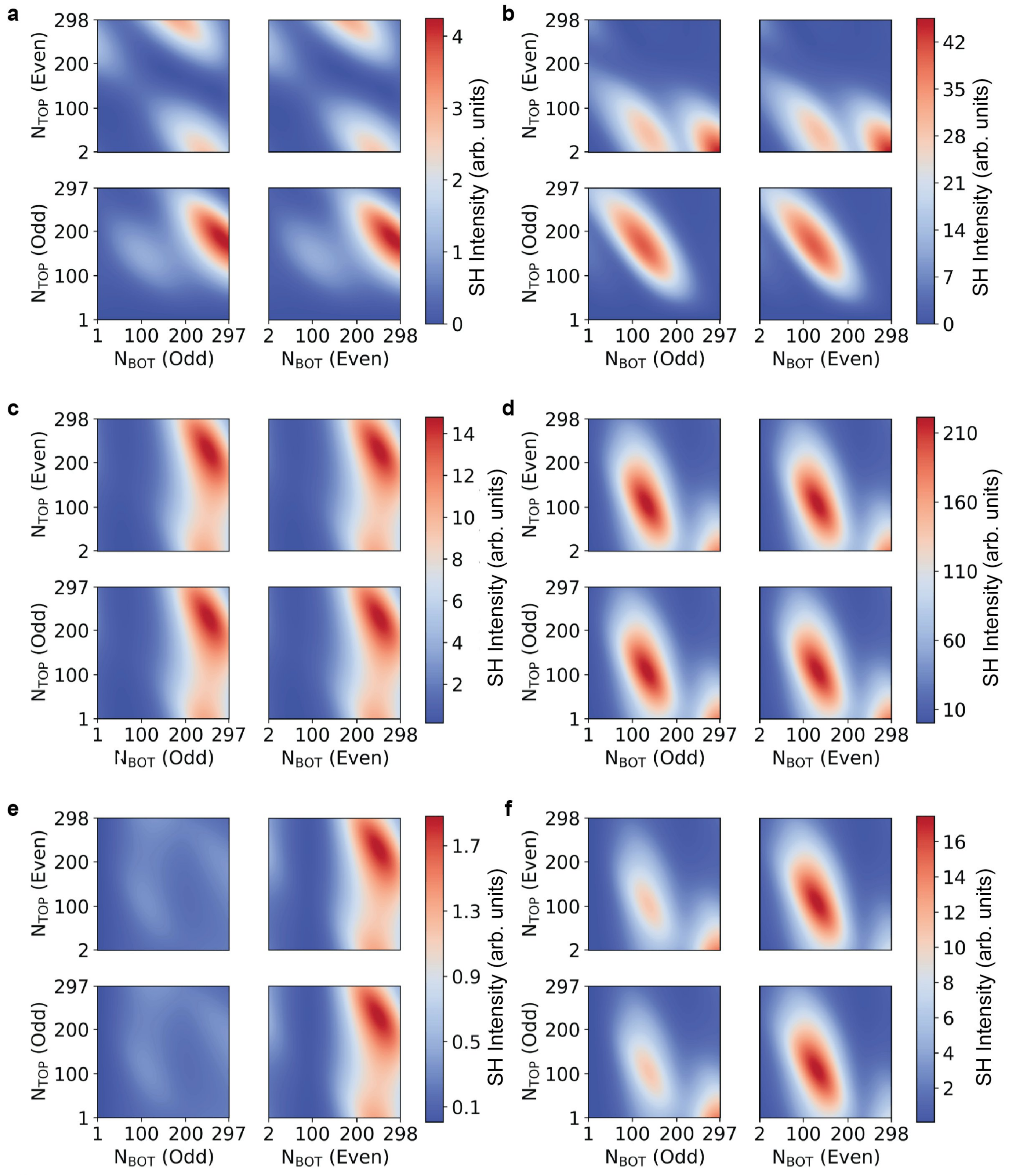

Supplementary Figure 9 | Isolated contributions to the SHG Signal from various components of twisted h-BN homostructures on different substrates. a, Contribution from the top h-BN flake on SiO_2/Si substrate. **b,** Contribution from the top h-BN flake on gold substrate. **c,** Contribution from the interface between h-BN flakes on SiO_2/Si substrate. **d,** Contribution from the interface between h-BN flakes on gold substrate. **e,** Contribution from the bottom h-BN flake on SiO_2/Si substrate. **f,** Contribution from the bottom h- BN flake on gold substrate. The main contributions for AB structures come from the interfaces between the stacking layers rather than the bulk. The enhancement from the gold substrate is observed for both the bulk and the interfaces.

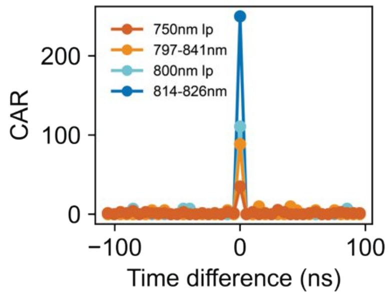

Supplementary Figure 10 | CAR with different filters. With narrower detection bandwidth, the value of CAR becomes higher.

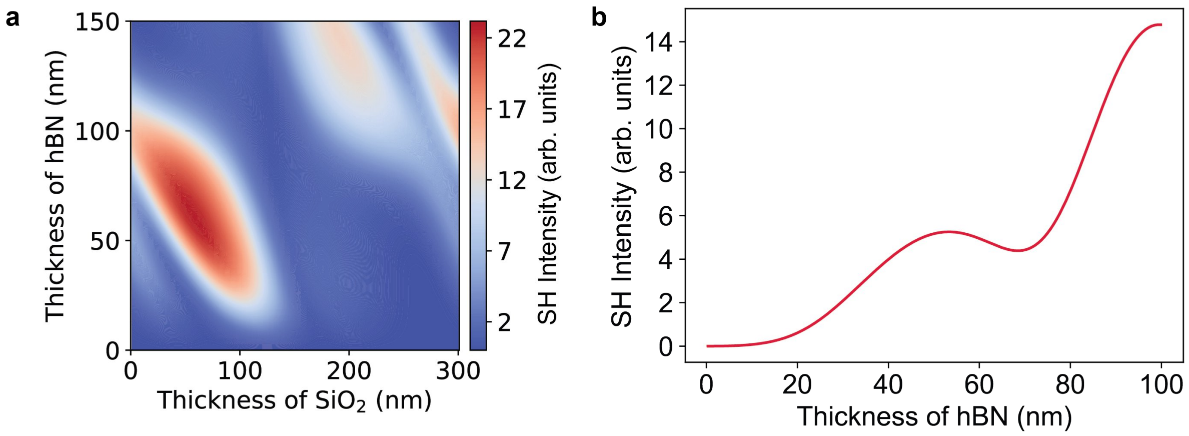

Supplementary Figure 11 | Simulation for SH Intensity emanating from even-layered h-BN on a S-A structure and gold film. Utilizing nonlinear transfer matrix calculations, a marked enhancement can be seen in signal strength for h-BN flakes comprising an even number

of monolayers on S-A structure (a), when compared to the results obtained on the gold film (b). The simulation corresponding to odd number of layers is shown as Figure 5b in the main text.

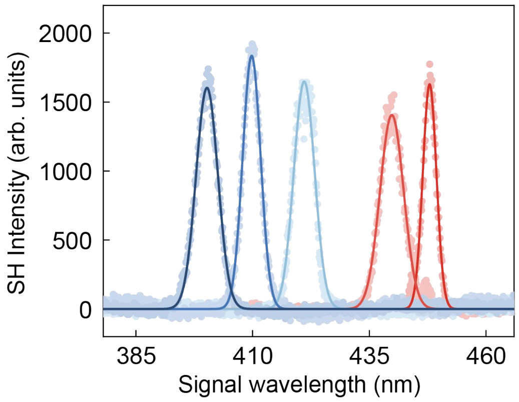

Supplementary Figure 12 | Wavelength dependence of SHG measurement from 8 nm h-BN on a SiO_2/Au configuration, where the SiO_2 thickness is 64 nm.

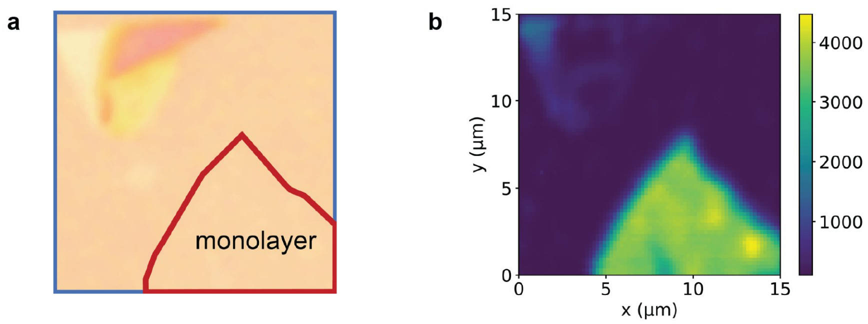

Supplementary Figure 13 | Innovative parity analysis of thick h-BN flakes using second harmonic generation. a, Optical microscope image of a 17 nm h-BN flake (in the blue box) on a S-A substrate with an extra monolayer transferred onto it (in the red curve). **b,** Nonlinear response map showcasing the contrast between the original 17 nm h-BN flake and after the addition of a monolayer, confirming the flake's even-layered structure.

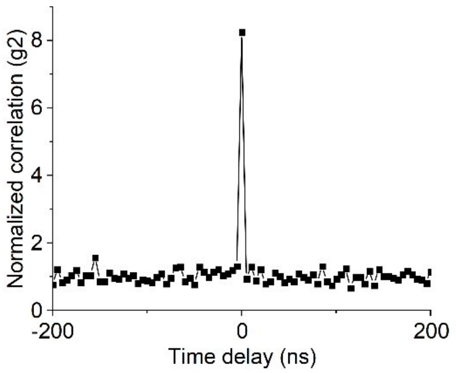

Supplementary Figure 14 | Correlation measurements on a 16 nm $NbOCl_2$ flake. The correlation measurement shows a peak of 8 when the time delay between two channels is 0 ns.

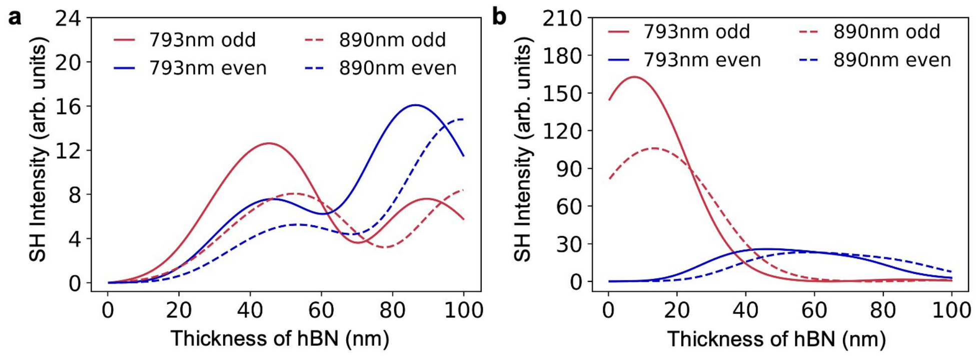

Supplementary Figure 15 | Simulations of the spectral dependence of the SHG response. The simulation results of SH intensity from h-BN with odd (red) and even (blue) layers on gold film (a) and S-A structure (b) as a function of thickness with 793 nm (solid line) and 890 nm (dashed line) pump field.

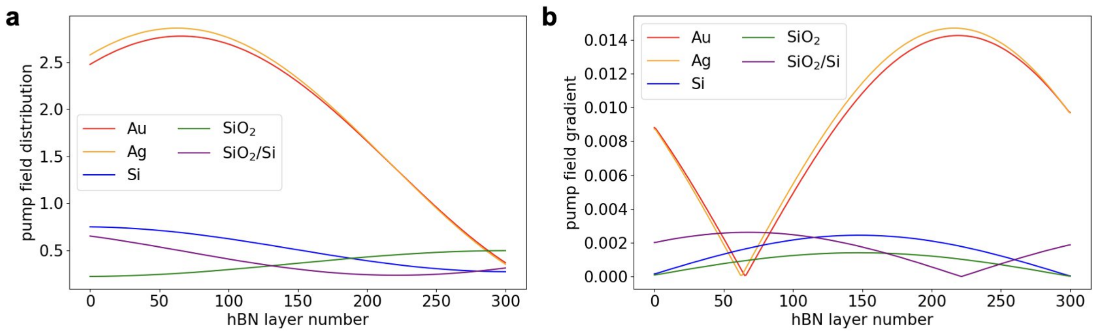

Supplementary Figure 16 | Pump field distribution and gradient inside h-BN flakes on different substrates. a, The metallic substrates (both Au and Ag) provide a significant higher

pump field enhancement, which contributes to the dipolar moments in the nonlinear response. **b,** The metallic substrates (both Au and Ag), because of the boundary condition imposed on the electric field by the metal at the h-BN/metal interface, provide a significant higher pump field gradient, which contributes to the quadrupolar moment in the nonlinear response.

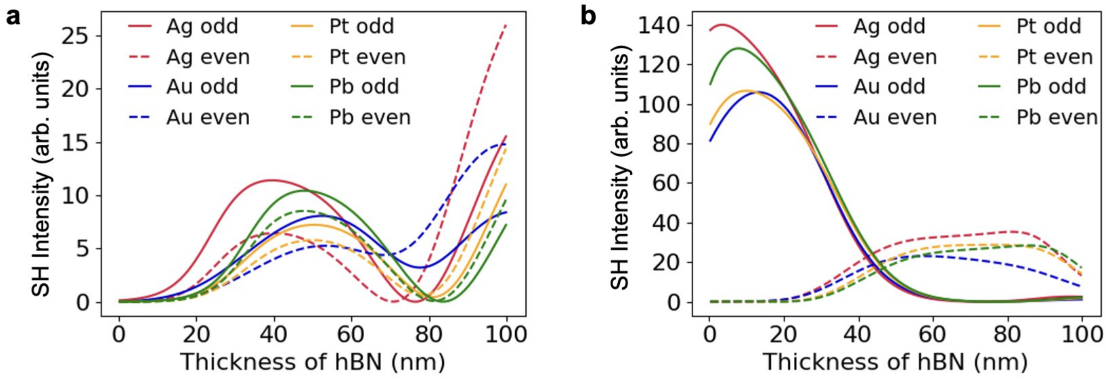

Supplementary Figure 17 | Simulations of SHG intensity as a function of thickness of h-BN from flakes on metallic (a) and SiO_2/metallic stack (b) substrates, where the SiO_2 layer has a thickness of 64 nm.

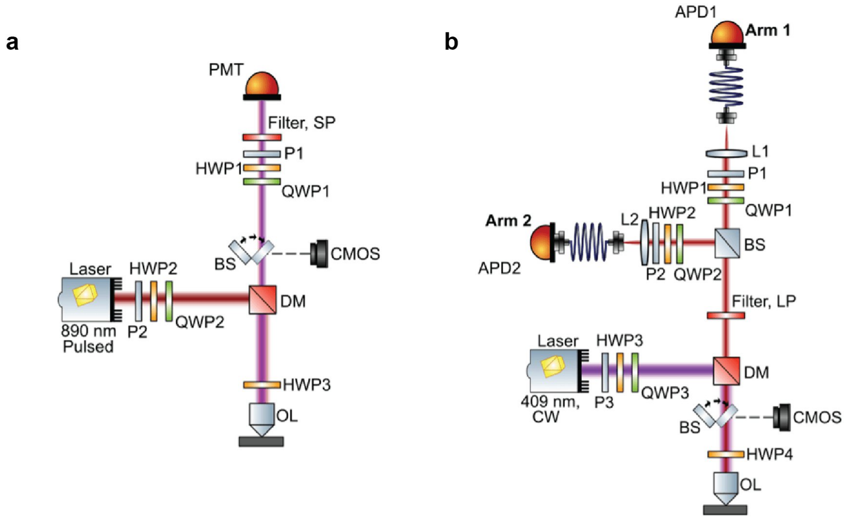

Supplementary Figure 18 | Schematic of optical setup. a, The schematic of SHG setup. **b,** The schematic of SPDC setup. PMT: photomultiplier tube. LP: longpass. SP: shortpass. P: polarizer. HWP: half waveplate. QWP: quarter waveplate. BS: beam splitter. DM: dichroic mirror. OL: objective lens. CW: continuous wave.

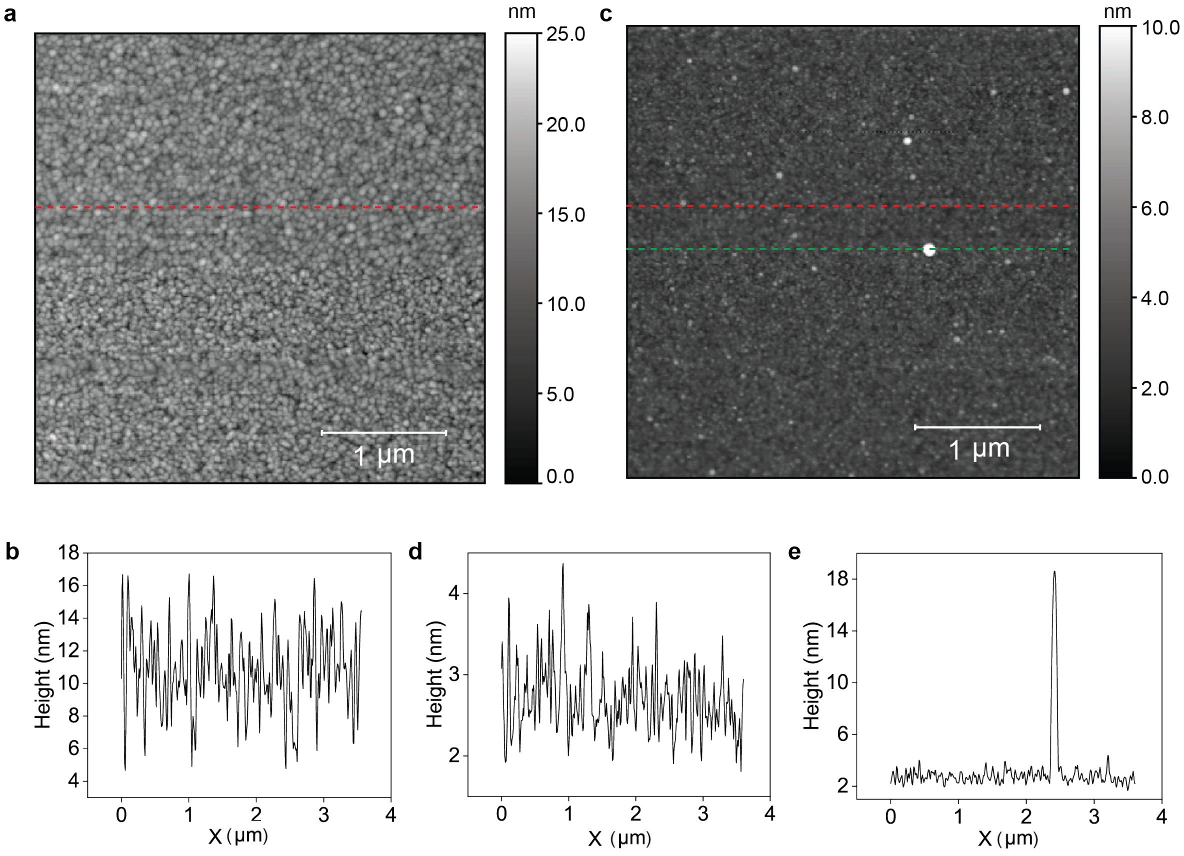

Supplementary Figure 19 | Surface morphology. a, AFM image of gold film. **b,** Height profile of the gold film along the red line indicated in (a). **c,** AFM image of SiO_2 film. **d,e,** Height profile of the SiO_2 film along the red(d) and green(e) line indicated in (c). Root mean square roughness is defined as $\sqrt{\overline{(h-\bar{h})^2}}$, where h is the height in each pixel.

Supplementary Tables

Supplementary Table 1 | Comparison of enhancement factor and operation bandwidth among different cavities and metasurfaces with relatively low Q factor.

Material	Structure	Enhancement factor	Operation bandwidth	Q factor	Ref.
a-Se	MS	100	340 nm – 360 nm	175	[1]
Lithium Niobate	MS	80	470 nm – 480 nm	75	[2]
poly-$BaTiO_3$	MS	16	350 nm – 550 nm	-	[3]
hBN	BIC	100	Narrow	300	[4]
MoS_2	MS	-	690 nm – 735 nm; 825 nm – 950 nm	low	[5]
MoS_2	nanodisk	1-23	400 nm – 500 nm	low	[6]

Supplementary Table 2 | Comparison of entangled photon pair sources presented in previous studies.

Nonlinear material	$\chi^{(2)}$ (pm/V)	Ref.
hBN	42	This work
$NbOCl_2$	400	This work
GaAs	119	[7]
BBO	2	[8]
MoS_2	300	[9]
WS_2	300	[9]
GaSe	30	[10]
$PdSe_2$	51.7	[11]
$LiNbO_3$	40	[12]

Supplementary Table 3 | Enhancement of SHG with cavities or metasurfaces.

Material	Structure	Enhancement factor	Operation wavelength	Ref.
Lithium Niobate	MS	80	470 nm – 480 nm	[2]
WS_2	gratings	400	860 nm	[13]
WS_2	MS	1140	832 nm	[14]
MoS_2	microcavity	10	818 nm	[15]
MoS_2	MS	35	850 nm	[16]
WS_2	MS	100-1000	810 nm	[17]
MoS_2	nanowire	140	800 nm	[18]
WS_2	MS	10	1240 nm	[19]
hBN	BIC	100	Tunable	[4]

Supplementary Table 4 | Comparison of active area thickness of quantum photon pair sources.

Photon pair source	Active area thickness (nm)	Ref.
AlGaAs (Mie-type)	400	[20]
LN (QOM)	680	[21]
GaAs (quasi BIC)	230	[22]
$LiNbO_3$ (silica meta-grating)	304	[23]
GaP	400	[24]
3R-MoS_2	285	[25]
3R-WS_2	350	[26]
$NbOCl_2$	46	[27]
$NbOCl_2$	1200	[28]
$NbOCl_2$	16	This work

Supplementary Table 5 | Reflectivity and transmission rate at different interfaces.

Interface	n_i	n_j	Reflection coefficient r	Reflectivity R	Transmission T
h-BN/Au	2.12	0.13+5.66i	-0.7428-0.6469i	0.97	0.03
h-BN/SiO_2	2.12	1.47	0.18	0.03	0.97
SiO_2/Si	1.47	3.67	-0.43	0.18	0.82

Supplementary Table 6 | Summary of the key characteristics of different structures, including nonlinear enhancement factors, physical mechanisms, benefits, and limitations.

Structures	Parity of nonlinear material layers	Process	Enhancement factors compared to SiO_2/Si	Physical mechanisms	Benefits	Limitations
h-BN/Au	Odd	SHG	Up to 738	Dipole, Quadrupole	Boadband excitation; Capable of other enhancements (e.g., twisted structures); Large-area enhancement	Not suitable for few-layer materials
h-BN/Au	Even	SHG	Up to 27	Quadrupole		
$NbOCl_2$/Au		SPDC	10	Dipole, Quadrupole		
Twisted h-BN/Au	-	SHG	357	Symmetry breaking, Dipole, Quadrupole		-
h-BN/64nm SiO_2/Au	Odd	SHG	238	Dipole, Quadrupole		Requires a specific SiO_2 thickness for optimal enhancement
$NbOCl_2$/ SiO_2/Au	-	SHG	300	Dipole, Quadrupole		
$NbOCl_2$/ SiO_2/Au	-	SPDC	Up to 100	Dipole, Quadrupole		

Supplementary Notes

Supplementary Note 1: Second harmonic susceptibility

Our selection of h-BN and $NbOCl_2$ is intentional, as they represent two distinct nonlinear regimes. In centrosymmetric materials like h-BN, the second-order susceptibility is inherently constrained by its structure (Supplementary Table 2). For even-layered h-BN, dipolar contributions vanish due to symmetry constraints, making the quadrupolar contribution dominant. This highlights the potential of methods designed to enhance quadrupolar nonlinear processes. Conversely, in non-centrosymmetric materials like $NbOCl_2$, the dipolar contribution is typically dominant, and the second-order susceptibility is intrinsically larger (Supplementary Table 2). Our method effectively enhances this response, similar to approaches using microcavities or metasurfaces. Additionally, our approach provides broadband enhancement, which is particularly beneficial for ultra-thin entangled photon sources such as $NbOCl_2$. This broadband response enables the generation of frequency-entangled photon pairs, thereby increasing the number of available quantum communication channels from a single source. In our simulations, we used a second-order susceptibility of 42 pm/V for h-BN and 400 pm/V for $NbOCl_2$. To further facilitate a direct comparison with existing literature, we have benchmarked our nonlinear materials by comparing their second-order susceptibility values, as presented in Supplementary Table 2.

Supplementary Note 2: Comparison of enhancement factor and operation bandwidth among different cavities and metasurfaces with relatively low Q factor

Tapajyoti et al. reported that significant SHG can be achieved with amorphous Selenium (Se)-based chalcogenide metasurfaces by exploiting the coupling between lattice and individual particle resonances [1]. At the resonant wavelength of $\lambda = 700$ nm, where field

enhancement is maximal, the SHG signal normalized to the incident power shows a 100-fold increase over the nonresonant region ($\lambda = 800$ nm). The quality factor of this structure is 175. With an increased period of 470 nm and the same particle size of 350 nm, a two-fold reduction of the field intensity and SHG emissions is observed, demonstrating broader resonances with lower quality factors.

Using lithium niobate (LN) membrane metasurfaces, Lun et al. achieved a SHG efficiency of 2.0×10^{-4}, which is significantly higher than previous LN implementations, with a narrow operational bandwidth of 10 nm and a Q factor of 75 [2]. Alternatively, Lucca et al. demonstrated high-Q resonances across the visible spectrum using quasi-bound states in the continuum (qBICs) on h-BN and leading to a 100-times enhancement [4], though this requires adjustments in resonator shapes for each wavelength. Mudassar et al. reported broad wavelength-dependent SHG enhancements in MoS_2 metasurfaces (690 nm - 735 nm; 825 nm - 950 nm) without specifying the enhancement factors [5]. Flavia et al. demonstrated a nonlinear optical response over a broad spectral range of 350 nm to 550 nm using a new type of metal oxide barium titanate-based nonlinear metasurface [3]. However, the absolute enhancement peaks at a relatively low value of 16. Anna et al. have reported MoS_2 nanodisks engineered to couple Mie resonances with C-excitons, which enables 23-fold enhancement of SHG intensity compared to monolayer MoS_2 [6]. This enhancement, while covering a broad wavelength range, is strongly wavelength-dependent and peaks at a 23-fold increase around 900 nm. The figures of merit discussed above have been summarized in Supplementary Table **1**. From the fundamental point of view (i.e. beyond the pure quantitative benchmark), the use of metal-based heterostructures exploits not only the enhanced electric-field intensity, as the photonic structures presented in the previous studies, but also the enhanced electric field gradient, induced by the zero electric field imposed by metals close to their surfaces.

Supplementary Note 3: Comparison of SHG enhancement factors enabled by coupling to resonant photonic structures

Nils et al. report that a WS_2 monolayer combined with a silicon metasurface hosting bound states in the BICs exhibits more than 3-orders of magnitude enhancement in second-harmonic intensity compared to a WS_2 monolayer on a flat silicon film of the same thickness [14]. However, this enhancement is limited to a narrow range around the resonance wavelength of 832 nm. Jared et al. reported enhanced SHG from monolayer MoS_2 embedded within an all-dielectric Fabry-Perot microcavity that is resonant at the pump wavelength 818 nm [15]. More works that focus on enhancement factor instead of bandwidth are summarized in Supplementary Table **3**. In contrast, our experiment demonstrates SHG enhancement over a much broader spectral range, exceeding 100 nm, with excitation wavelengths from 790 nm to 890 nm. While the range is currently constrained by the operating wavelength of our laser, we believe that the actual bandwidth could extend beyond 100 nm.

Supplementary Note 4: SHG, SFG and SPDC

For classical processes such as SHG and SFG (sum frequency generation), energy and phase matching conditions are stringent, typically resulting in photon generation at specific frequencies determined by the pump frequencies. For the quantum SPDC process, the total energy of the photon pairs (signal and idler) must sum to the pump photon energy. However, the frequencies of the individual photons are not fixed and can vary across a broad spectrum, due to the relaxed phase-matching conditions in thin film. The classical estimation of the SPDC generation rates can be related to the SFG process by: $\frac{1}{\Phi_p}\frac{dN_{pair}}{dt} = 2\pi\Xi^{SFG}\frac{\lambda_p^4}{\lambda_s^3\lambda_i^3}\frac{c\Delta\lambda}{\lambda_s^2}$, where Φ_p is the SPDC pump flux, $\lambda_{x=p,s,i}$ are the pump, signal, idler wavelengths and $\Delta\lambda$ is the nonlinear resonance bandwidth at the signal/idler wavelengths. Therefore, SPDC is not appropriately described by a simple proportional relationship to SHG, but related to the integration of all possible SFG processes over the whole spatial and spectral spectrum.

Supplementary Note 5: Dipole moment and Quadruple moment

In hBN, the polarization arises from two components: $P_\alpha^{2\omega} = P_\alpha^{2\omega,\mathrm{d}} + P_\alpha^{2\omega,\mathrm{q}}$, where $P_\alpha^{2\omega,d}$ is the dipole moment and $P_\alpha^{2\omega,q}$ is the quadrupole moment. These are expressed as

$$P_\alpha^{2\omega,\mathrm{d}} = \varepsilon_0 \chi_{\alpha\beta\gamma}^{(2)} E_\beta^\omega E_\gamma^\omega \tag{1}$$

$$P_\alpha^{2\omega,\mathrm{q}} = \varepsilon_0 \chi_{\alpha\beta\gamma}^{(4)} E_\beta^\omega \frac{\partial E_\gamma^\omega}{\partial z} \tag{2}$$

where the subscripts α, β, γ denoting in-plane polarization directions in Cartesian coordinates, E^ω is the pump field. In monolayers of hBN, governed by D_{3h} point group symmetry, the monolayer quadrupole moments $P_\alpha^{2\omega,q}$ vanish due to the reflection symmetry, rendering $\chi_{\alpha\beta\gamma}^{(4),1L} = 0$. However, in bilayer configurations, where reflection symmetry is broken, a nonzero quadrupole moment emerges. This arises from the gradient in the electric field across adjacent layers. Consider nonlinear polarization from a bilayer unit including layer j and j + 1 is, where the dipole moment vanishes due to centrosymmetry leaving the quadrupole moment as the leading term:

$$P_\alpha^{2\omega,q,2L} = P_\alpha^{2\omega,d,j} + P_\alpha^{2\omega,d,j+1} = \varepsilon_0 \chi_{\alpha\beta\gamma}^{(2)} E_\beta^{\omega,j+1} E_\gamma^{\omega,j+1} - \varepsilon_0 \chi_{\alpha\beta\gamma}^{(2)} E_\beta^{\omega,j} E_\gamma^{\omega,j} \tag{3}$$

$$= \varepsilon_0 \chi_{\alpha\beta\gamma}^{(2)} \left(E_\beta^{\omega,j} + \Delta E_\beta^\omega\right) \left(E_\gamma^{\omega,j} + \Delta E_\gamma^\omega\right) - \varepsilon_0 \chi_{\alpha\beta\gamma}^{(2)} E_\beta^{\omega,j} E_\gamma^{\omega,j} \tag{4}$$

$$\approx 2\varepsilon_0 \chi_{\alpha\beta\gamma}^{(2)} d_{BN} E_\beta^{\omega,j} \frac{\partial E_\gamma^\omega}{\partial z} \tag{5}$$

where d_{BN} is the distance between the adjacent two layers. We can see the effective quadrupole susceptibility $\chi_{\alpha\beta\gamma}^{(2),2L,q} = 2\chi_{\alpha\beta\gamma}^{(2),d} d_{BN} \neq 0$, which also indicates that the tensor symmetries of the quadrupolar and dipolar terms are identical under these conditions [29].

Now let us consider even layer h-BN and odd layer h-BN separately:

For the even parity h-BN: Even-layer material that owns the centrosymmetry of bulk material can still show a weak SHG signal. This is all attributed to the nonzero quadrupolar

moment since the dipole moment vanish due to the centrosymmetry. In our method, we have enhanced the quadrupolar moment by increasing the gradient of the electric field $\frac{\partial E_{\gamma}^{\omega}}{\partial z}$as well as by amplifying $E_{\beta}^{\omega,j}$within the material (both contribution (i) and (ii)). This enhancement is demonstrated in Figure 2a of the main text.

For the odd parity h-BN: Both the dipole and quadrupolar moments contribute to the SHG signal. This system includes a combination of two adjacent layers, akin to the even-layer material, and an additional single layer. In the two-layer configuration, despite the absence of a dipole moment (due to symmetry cancellation), the quadrupole moment remains nonzero (as discussed in (1)). Conversely, in the single-layer configuration, the dipole moment is present, but the quadrupole moment is zero. Thus, the overall nonlinear polarization $P_{\alpha}^{2\omega}$ can be expressed as $P_{\alpha}^{2\omega,d,SL} + P_{\alpha}^{2\omega,q,2L}$, where SL denotes one single layer and 2L denotes two adjacent layers. By placing h-BN on a gold substrate, enhancements are observed in all of E_{β}^{ω}, E_{γ}^{ω} and $\frac{\partial E_{\gamma}^{\omega}}{\partial z}$ (both contribution (i) and (ii)), effectively boosting both dipole and quadrupole contributions. This enhancement is depicted in Supplementary Figure 2, where a and b illustrate the effects on the SiO_2/Si substrate, and c and d demonstrate the enhancements with the gold substrate. As shown in the figures, with gold as the substrate, the quadrupolar response in h-BN is only 2 to 3 times smaller than the dipolar response. This close magnitude between the two contributions underscores the significant impact of the quadrupolar moment in altering the nonlinear response of a material, especially in centrosymmetric materials where the quadrupolar contribution is dominant.

Supplementary Note 6: Effect of SiO_2 Thickness on the SHG Enhancement

Regarding the influence of substrate thickness on the enhancement factor, we performed additional simulations to systematically examine the effect of varying SiO_2 layer thickness on the SHG response for material/SiO_2/X structures, where X represents metals (Au, Pt, Ag, Pb) as well as silicon (Si). The simulations also included a comparison with pure

SiO_2 (fused silica). Our analysis, covering multiple hBN thicknesses, revealed that the thickness of the SiO_2 layer indeed influences the SHG signal due to interference effects. In our experiment, we used a commercial wafer, where the thickness of SiO_2 is 285 nm. Structures involving material/SiO_2/metal combinations outperformed those with SiO_2/Si and pure SiO_2 substrates even with optimal thickness of SiO_2 (Supplementary Figure 1). This result underscores the critical role of interference and reflection effects in enhancing the nonlinear response, with material/SiO_2/metal structures showing consistent performance improvement across different configurations.

Additionally, we explored the SHG responses for odd- and even-layer hBN separately, emphasizing the role that metals play in enhancing the electric field gradient. This enhancement contributes to quadruple moments, which are particularly important for centrosymmetric materials like even-layer hBN, allowing them to exhibit nonzero SHG signals.

Supplementary Note 7: Innovative parity analysis of thick h-BN flakes using second harmonic generation

We intentionally demonstrate the capability of our method to resolve subtle differences in layer numbers using SHG measurements. Specifically, we illustrate how this approach enables a clear distinction between N-monolayer and N+1-monolayer h-BN, even when N is large. This capability is particularly advantageous over standard characterization on SiO_2 /Si substrates, where such precision becomes challenging as the number of layers increases. To validate this, we monitored the SH photon count rate before and after transferring a monolayer flake onto a thick 17 nm h-BN layer (Supplementary Figure 13a.). The SHG intensity measurement, shown in Supplementary Figure 13b, reveals a contrast between the original flake and its augmented state following the monolayer addition. This pronounced difference confirms the even-layered structure of the underlying flake, consistent with our theoretical simulations.

This versatility arises from the significant enhancement in SHG intensity observed for

h-BN layers on our engineered substrates. As discussed in the main text (Figure 5e), the SHG signal is enhanced by up to three orders of magnitude compared to that of h-BN on conventional SiO_2/Si substrates. This demonstration highlights the practicality of our method for the 2D materials community, enabling precise parity analysis of h-BN samples, even for thick structures.

Supplementary Note 8: Broadband SHG on SiO_2/Au configuration

We have investigated the broadband capabilities of the SiO_2/Au/SiO_2/Si structure. Our findings indicate that the SiO_2/Au/SiO_2/Si substrate maintains robust broadband characteristics, effectively supporting a wide range of wavelengths without significant losses in field enhancement, as illustrated in the Supplementary Figure 12. This broad bandwidth can be attributed to the subwavelength thickness of the active nonlinear material.

Supplementary Note 9: Nonlinear transfer matrix method

In this paper the assumption is made that the nonlinear processes occurring in the h-BN material are much weaker compared to the pump field and, thus, the pump is not depleted across the h-BN due to the nonlinear process itself. Building upon the established theory for third harmonic generation (THG) [30], we calculate the SHG of h-BN on various structures. The analysis of the nonlinear optical process follows a systematic approach: First, the distribution of the pump field inside the structures is simulated. Next, the nonlinear polarization components for each layer are calculated. Subsequently, the nonlinear polarizations are treated as sources, and the propagation of the second harmonic signals across all the layers is determined. Finally, the contributions of the individual layers are summed up, resulting in the overall SHG signal.

Consider a wave traveling in a layered material in z-direction with an electric field written

as $E(z) = E^{+}(z) + E^{-}(z)$ for both forward- and backward-propagating, where

$$E(z,t) = E^{+}e^{(ink_0 d)+(ik_z z - i\omega t)} + E^{-}e^{(-ink_0 d)+(ik_z z - i\omega t)} \tag{6}$$

where n is the refractive index of the medium, which is a complex number with its imaginary part positive when the material absorbs light, k_0 is the wave-vector magnitude in the vacuum, d is the traveling distance of wave, ω is the frequency of the wave. In our analysis, we consider the incident light to be normal to the surface of the sample, aligned along the z-direction. Under this configuration, both the s-polarization and p-polarization components exhibit the same expressions and behavior. The electric field can also be written in the matrix form:

$$E(z) = \begin{bmatrix} E_i^{+}(z) \\ E_i^{-}(z) \end{bmatrix} \tag{7}$$

Fresnel's equations allow us to calculate the intensity and phase changes of the reflected and transmitted light at the interface based on the refractive indices of the two media:

$$r_{ij} = \frac{n_i - n_j}{n_i + n_j} \tag{8}$$

$$t_{ij} = \frac{2n_i}{n_i + n_j} \tag{9}$$

where n_i and n_j describe the refractive indexes of medium 1 and 2, respectively. It is not difficult to get:

$$r_{ij} = -r_{ji}, t_{ji} = \frac{1 - r_{ij}^2}{t_{ij}} \tag{10}$$

The relationship between the electric fields in media 1 and 2 can be described by the reflection and transmission matrices:

$$\begin{bmatrix} E_i^{+} \\ E_i^{-} \end{bmatrix} = \mathbf{M}_{ij} \begin{bmatrix} E_j^{+} \\ E_j^{-} \end{bmatrix} \tag{11}$$

with

$$\mathbf{M}_{ij} = \frac{1}{t_{ij}} \begin{bmatrix} 1 & r_{ij} \\ r_{ij} & 1 \end{bmatrix} \tag{12}$$

which is the transfer matrix from medium 1 to medium 2, and it has the property $\mathbf{M}_{ij}^{-1} = \mathbf{M}_{ji}$.

To express the right and left propagating wave at a distance d in the matrix formulation we employ:

$$\begin{bmatrix} E_i^+ \\ E_i^- \end{bmatrix} = \begin{bmatrix} e^{-ink_0d} & 0 \\ 0 & e^{ink_0d} \end{bmatrix} \begin{bmatrix} E_d^+ \\ E_d^- \end{bmatrix} \tag{13}$$

Here the matrix $\bar{\Phi}_i = \begin{bmatrix} e^{-ink_0d} & 0 \\ 0 & e^{ink_0d} \end{bmatrix}$ is called the propagation matrix, and it is straightforward to notice that $\bar{\Phi}_i = \bar{\Phi}_i^{-1}$ when light is incident from right.

The overall transfer matrix for a plane wave light from left to right in a multi-layer system now can be written as:

$$\mathbf{T} = \mathbf{M}_{12}\Phi_2\mathbf{M}_{23}\Phi_3 \cdots \mathbf{M}_{ij}\Phi_j \cdots \Phi_{f-1}\mathbf{M}_{(f-1)f} \tag{14}$$

So the relationship between the wave at the left boundary of the structure and the wave at the right boundary of the structure is given by the overall transfer matrix:

$$\mathbf{E}_0 = \mathbf{T}\mathbf{E}_f = \begin{bmatrix} \mathbf{T}_{00} & \mathbf{T}_{01} \\ \mathbf{T}_{10} & \mathbf{T}_{11} \end{bmatrix} \mathbf{E}_f \tag{15}$$

Consider the light is incident from left and there is no back-propagating light from the right boundary, which means $\mathbf{E_0} = \begin{bmatrix} E_0^+ \\ 0 \end{bmatrix}$ and $\mathbf{E_f} = \begin{bmatrix} E_f^+ \\ E_f^- \end{bmatrix}$. The reflection coefficient and transmission coefficient of the whole system are:

$$r = \frac{E_0^-}{E_0^+} = \frac{\mathbf{T}_{10}}{\mathbf{T}_{00}}, t = \frac{E_f^-}{E_0^+} = \frac{1}{\mathbf{T}_{11}} \tag{16}$$

Consider the light is incident from right and there is no back-propagating light from the left boundary, which means $\mathbf{E_0} = \begin{bmatrix} E_0^+ \\ E_0^- \end{bmatrix}$ and $\mathbf{E_f} = \begin{bmatrix} 0 \\ E_f^- \end{bmatrix}$. The coefficients are:

$$r = \frac{E_f^-}{E_f^+} = -\frac{\mathbf{T}_{00}}{\mathbf{T}_{01}}, t = \frac{E_0^-}{E_f^-} = \frac{\mathbf{T}_{00}\mathbf{T}_{11} - \mathbf{T}_{01}\mathbf{T}_{10}}{\mathbf{T}_{00}} \tag{17}$$

The reflectivity and transmission of the light in this multi-layer structure can be calculated with $R = |r|^2$ and $T = |t|^2$.

For right propagating waves, the amplitudes of the wave at the left edge of layer i can be expressed as:

$$\begin{bmatrix} 1 \\ r \end{bmatrix} = \mathbf{T}_{1i} \begin{bmatrix} E_i^+ \\ E_i^- \end{bmatrix} \tag{18}$$

Once the pump field distribution at each layer has been determined, we can turn to compute the nonlinear polarization by treating each layer as a nonlinear source and sum the amplitude contributions from all the layers. Within layer j, the second harmonic nonlinear polarization can be expressed as:

$$P^j_{2\omega,\alpha} = \epsilon_0 \left|\chi^{(2)}_{\alpha\beta\gamma}\right| \cos\left(3\varphi^j\right) \left[E^{j,+}_{\omega,\beta} e^{ik_\omega z} + E^{j,-}_{\omega,\beta} e^{-ik_\omega z}\right] \left[E^{j,+}_{\omega,\gamma} e^{ik_\omega z} + E^{j,-}_{\omega,\gamma} e^{-ik_\omega z}\right] \times e^{i(k_{2x} - \omega_2 t)} \tag{19}$$

$\chi^{(2)}$ is the second-order susceptibility and the nonvanishing terms for h-BN are $\chi^{(2)} = \chi^{(2)}_{yyy} = -\chi^{(2)}_{yxx} = -\chi^{(2)}_{xxy} = -\chi^{(2)}_{xyx}$, which determines the dependence of polarization components on the sample orientation. ϕ^j is the angle between the polarization direction of pump field and the armchair direction in h-BN of layer j. By varying the angle, polarization dependent second harmonic generation (SHG) can be calculated. Note that $k_{2x} = 2k_x$ and $\omega_2 = 2\omega$. By fixing the pump field in the y direction, the polarization can be simplified as:

$$P_{2\omega,y} = \epsilon_0 \left|\chi^{(2)}\right| \cos\left(3\varphi^j\right) \left[E^{+}_{\omega,y} e^{ik_\omega z} + E^{-}_{\omega,y} e^{-ik_\omega z}\right]^2 \tag{20}$$

By considering first s-polarized free waves, the bound wave source is correspondingly given by:

$$\mathbf{E_S} = \frac{4\pi}{\epsilon_s - \epsilon_j}\mathbf{P}_y \tag{21}$$

where $\epsilon_j = \epsilon_{2\omega}$. According to Eq.20, we obtain the bound wave source expression with different wavevectors:

$$\mathbf{E}_S^{2k_\omega} = \frac{4\pi}{\epsilon_\omega - \epsilon_{2\omega}}\mathbf{P}_{2\omega}^{2k_\omega} \tag{22}$$

$$\mathbf{E}_S^{0} = -\frac{4\pi}{\epsilon_{2\omega}}\mathbf{P}_{2\omega}^{0} \tag{23}$$

with

$$\boldsymbol{P}_{2\omega}^{2k_\omega} = \epsilon_0 \left|\chi^{(2)}\right| \cos\left(3\varphi^j\right) \begin{bmatrix} E_\omega^{+2} \\ E_\omega^{-2} \end{bmatrix} \tag{24}$$

$$\boldsymbol{P}_{2\omega}^{0} = \epsilon_0 \left|\chi^{(2)}\right| \cos\left(3\varphi^j\right) \begin{bmatrix} E_\omega^{+} E_\omega^{-} \\ E_\omega^{-} E_\omega^{-} \end{bmatrix} \tag{25}$$

where we assume that the SH signal is not strong enough to deplete the pump field. The bound wave source $\mathbf{E}_S$ in layer j drives the free wave in the adjacent i, j, k layers, which also obey the transfer and propagation rules as discussed earlier:

$$\mathbf{E}_i = \mathbf{M}_{ij}\mathbf{E}_j + \mathbf{M}_{is}\mathbf{E}_s \tag{26}$$

$$\mathbf{E}_k = \mathbf{M}_{kj}\mathbf{\Phi}_j\mathbf{E}_j + \mathbf{M}_{ks}\mathbf{\Phi}_s\mathbf{E}_s \tag{27}$$

here we only consider the left edge of each layer for the bound wave. Given that $\mathbf{M}_{kj}\mathbf{M}_{js} = \mathbf{M}_{ks}$ and $\mathbf{M}_{ji}\mathbf{M}_{is} = \mathbf{M}_{js}$, (27) can be written as:

$$\mathbf{E}_k = \mathbf{M}_{kj}\mathbf{\Phi}_j(\mathbf{M}_{ji}\mathbf{E}_i + \mathbf{S}_j) \tag{28}$$

$$\mathbf{S}_j = (\bar{\mathbf{\Phi}}_j\mathbf{M}_{js}\mathbf{\Phi}_s - \mathbf{M}_{js})\mathbf{E}_S \tag{29}$$

and two different wavevectors terms become added together:

$$\mathbf{E}_k = \mathbf{M}_{kj}\mathbf{\Phi}_j(\mathbf{M}_{ji}\mathbf{E}_i + \mathbf{S}_j^{2k_\omega} + \mathbf{S}_j^0) \tag{30}$$

$$\mathbf{S}_j^{2k_\omega} = (\bar{\mathbf{\Phi}}_j\mathbf{M}_{js}^{2k_\omega}\mathbf{\Phi}_s^{2k_\omega} - \mathbf{M}_{js}^{2k\omega})\mathbf{E}_S^{2k_\omega} \tag{31}$$

$$\mathbf{S}_j^0 = (\bar{\mathbf{\Phi}}_j\mathbf{M}_{js}^0\mathbf{\Phi}_s^0 - \mathbf{M}_{js}^0)\mathbf{E}_S^0 \tag{32}$$

The propagation wavevector of matrices Φ for 2ω is $2k_\omega$ and the refractive indexes need to be reevaluated accordingly, which are $n^{2k_\omega} = n_\omega$ and $n^0 = 0$. S_j is the free-field amplitude for 2ω electric field. The amplitudes of 2ω fields now can be expressed as a function of $\mathbf{S}_0$ and $\mathbf{S}_{2k_\omega}$:

$$\begin{bmatrix} E_f^+(j) \\ E_0^-(j) \end{bmatrix} = \frac{1}{\bar{T}_{11}} \begin{bmatrix} 1 & 0 \\ \bar{T}_{21} & -\bar{T}_{11} \end{bmatrix} \mathbf{S}'_{j,2k_\omega} + \mathbf{S}'_{j,0} \tag{33}$$

with $\mathbf{S}'_j = \bar{\mathbf{L}}_{j1}\mathbf{S}$, $\bar{\mathbf{T}}$ is the total right to left transfer matrix and $\bar{\mathbf{L}}_{j1}$ is the transfer matrix from first layer to layer j. Finally, by summing the $E_0^-(j)$ over all the layers, the intensity of the total second-harmonic electric field can be written as:

$$I^{tot} = \left|E_{2\omega,0}^-\right|^2 = \left|\sum_{j=0}^{f} E_{2\omega,0}^-(j)\right|^2 \tag{34}$$

Supplementary References

1. Das Gupta, T. *et al.* Second harmonic generation in glass-based metasurfaces using tailored surface lattice resonances. *Nanophotonics* **10,** 3465–3475 (2021).
2. Qu, L. *et al.* Giant second harmonic generation from membrane metasurfaces. *Nano Letters* **22,** 9652–9657 (2022).
3. Timpu, F. *et al.* Enhanced nonlinear yield from barium titanate metasurface down to the near ultraviolet. *Advanced Optical Materials* **7,** 1900936 (2019).
4. Kühner, L. *et al.* High-Q nanophotonics over the full visible spectrum enabled by hexagonal boron nitride metasurfaces. *Advanced Materials* **35,** 2209688 (2023).
5. Nauman, M. *et al.* Tunable unidirectional nonlinear emission from transition-metal-dichalcogenide metasurfaces. *Nature communications* **12,** 5597 (2021).
6. Popkova, A. A. *et al.* Nonlinear exciton-mie coupling in transition metal dichalcogenide nanoresonators. *Laser & Photonics Reviews* **16,** 2100604 (2022).
7. Shoji, I., Kondo, T., Kitamoto, A., Shirane, M. & Ito, R. Absolute scale of second-order nonlinear-optical coefficients. *JOSA B* **14,** 2268–2294 (1997).
8. Okoth, C., Cavanna, A., Santiago-Cruz, T. & Chekhova, M. V. Microscale generation of entangled photons without momentum conservation. *Physical review letters* **123,** 263602 (2019).
9. Yao, K. *et al.* Continuous wave sum frequency generation and imaging of monolayer and heterobilayer two-dimensional semiconductors. *ACS nano* **14,** 708–714 (2019).
10. Jie, W. *et al.* Layer-dependent nonlinear optical properties and stability of non-centrosymmetric modification in few-layer GaSe sheets. *Angewandte Chemie International Edition* **54,** 1185–1189 (2015).
11. Yu, J. *et al.* Giant nonlinear optical activity in two-dimensional palladium diselenide. *Nature communications* **12,** 1083 (2021).

12. Ribeiro-Soares, J. *et al.* Second harmonic generation in WSe2. *2D Materials* **2,** 045015 (2015).

13. Shi, J. *et al.* Plasmonic enhancement and manipulation of optical nonlinearity in monolayer tungsten disulfide. *Laser & Photonics Reviews* **12,** 1800188 (2018).

14. Bernhardt, N. *et al.* Quasi-BIC resonant enhancement of second-harmonic generation in WS2 monolayers. *Nano Letters* **20,** 5309–5314 (2020).

15. Day, J. K., Chung, M.-H., Lee, Y.-H. & Menon, V. M. Microcavity enhanced second harmonic generation in 2D MoS 2. *Optical Materials Express* **6,** 2360–2365 (2016).

16. Löchner, F. J. *et al.* Hybrid dielectric metasurfaces for enhancing second-harmonic generation in chemical vapor deposition grown MoS2 monolayers. *Acs Photonics* **8,** 218–227 (2020).

17. Chen, J. *et al.* Tungsten disulfide–gold nanohole hybrid metasurfaces for nonlinear metalenses in the visible region. *Nano Letters* **18,** 1344–1350 (2018).

18. Li, D. *et al.* Anisotropic enhancement of second-harmonic generation in monolayer and bilayer MoS2 by integrating with TiO2 nanowires. *Nano Letters* **19,** 4195–4204 (2019).

19. Hu, G. *et al.* Coherent steering of nonlinear chiral valley photons with a synthetic Au–WS2 metasurface. *Nature Photonics* **13,** 467–472 (2019).

20. Marino, G. *et al.* Spontaneous photon-pair generation from a dielectric nanoantenna. *Optica* **6,** 1416–1422 (2019).

21. Santiago-Cruz, T. *et al.* Photon pairs from resonant metasurfaces. *Nano letters* **21,** 4423–4429 (2021).

22. Santiago-Cruz, T. *et al.* Resonant metasurfaces for generating complex quantum states. *Science* **377,** 991–995 (2022).

23. Zhang, J. *et al.* Spatially entangled photon pairs from lithium niobate nonlocal metasurfaces. *Science Advances* **8,** eabq4240 (2022).

24. Sultanov, V., Santiago-Cruz, T. & Chekhova, M. V. Flat-optics generation of broadband photon pairs with tunable polarization entanglement. *Optics Letters* **47,** 3872–3875 (2022).

25. Weissflog, M. A. *et al.* A tunable transition metal dichalcogenide entangled photon-pair source. *Nature Communications* **15,** 7600 (2024).

26. Feng, J. *et al.* Polarization-entangled photon-pair source with van der Waals 3R-WS2 crystal. *eLight* **4,** 16 (2024).

27. Guo, Q. *et al.* Ultrathin quantum light source with van der Waals NbOCl2 crystal. *Nature* **613,** 53–59 (2023).

28. Kallioniemi, L. *et al.* Van der Waals engineering for quantum-entangled photon generation. *Nature Photonics,* 1–7 (2024).

29. Yao, K. *et al.* Enhanced tunable second harmonic generation from twistable interfaces and vertical superlattices in boron nitride homostructures. *Science Advances* **7,** eabe8691 (2021).

30. Bethune, D. Optical harmonic generation and mixing in multilayer media: analysis using optical transfer matrix techniques. *JOSA B* **6,** 910–916 (1989).
